# Obstetric anesthesia services in Israel snapshot (OASIS) study: a 72 hour cross-sectional observational study of workforce supply and demand

**DOI:** 10.1186/s13584-021-00460-2

**Published:** 2021-03-15

**Authors:** Gal Schtrechman-Levi, Alexander Ioscovich, Jacob Hart, Jacob Bar, Ronit Calderon-Margalit, Eshel A. Nir, Yehuda Ginosar

**Affiliations:** 1 Department of Epidemiology and Preventive Medicine, School of Public Health, Sackler School of Medicine, Tel Aviv University, Tel Aviv-Yafo, Israel; 2 Department of General and Oncological Surgery – Surgery C, The Haim Sheba Medical Center, Ramat Gan, Israel; 3 Department of Anesthesiology, Perioperative Medicine, and Pain Treatment, Shaare Zedek Medical Center, Jerusalem, Israel; 4 Health Services Management School, Netanya Academic College, Netanya, Israel; 5 Department of Obstetrics and Gynecology, Wolfson Medical Center, Holon, Israel; 6 Hadassah-Hebrew University Braun School of Public Health, Jerusalem, Israel; 7 Faculty of Medicine, Hebrew University of Jerusalem, Israel, Department of Anesthesia and Operating Rooms, Kaplan Medical Center, Rehovot, Israel; 8 Department of Anesthesiology and Critical Care Medicine, Wohl Institute of Translational Medicine, Hadassah-Hebrew University Medical Center, Jerusalem, Israel; 9 Department of Anesthesiology, Washington University School of Medicine, St Louis, MO USA

**Keywords:** Obstetric anesthesia, Health service, Workload, Workforce, Epidural analgesia, Cesarean section

## Abstract

**Background:**

We planned an observational study to assess obstetric anesthesia services nationwide. We aimed to assess the effect of the anesthesia workload/workforce ratio on quality and safety outcomes of obstetric anesthesia care.

**Methods:**

Observers prospectively collected data from labor units over 72 h (Wednesday, Thursday and Friday). Independent variables were workload (WL) and workforce (WF). WL was assessed by the Obstetric Anesthesia Activity Index (OAAI), which is the estimated time in a 24-h period spent on epidurals and all cesarean deliveries. Workforce (WF) was assessed by the number of anesthesiologists dedicated to the labor ward per week. Dependent variables were the time until anesthesiologist arrival for epidural (quality measure) and the occurrence of general anesthesia for urgent Cesarean section, CS, (safety measure). This census included vaginal deliveries and unscheduled (but not elective) CS.

**Results:**

Data on 575 deliveries are from 12 maternity units only, primarily because a major hospital chain chose not to participate; eight other hospitals lacked institutional review board approval. The epidural response rate was 94.4%; 321 of 340 parturients who requested epidural analgesia (EA) received it. Of the 19 women who requested EA but gave birth without it, 14 (77%) were due to late arrival of the anesthesiologist. Median waiting times for anesthesiologist arrival ranged from 5 to 28 min. The OAAI varied from 4.6 to 25.1 and WF ranged from 0 to 2 per shift. Request rates for EA in hospitals serving predominantly orthodox Jewish communities and in peripheral hospitals were similar to those of the entire sample. More than a fifth (13/62; 21%) of the unscheduled CS received general anesthesia, and of these almost a quarter (3/13; 23%) were attributed to delayed anesthesiologist arrival.

**Conclusions:**

Inadequate WF allocations may impair quality and safety outcomes in obstetric anesthesia services. OAAI is a better predictor of WL than delivery numbers alone, especially concerning WF shortage. To assess the quality and safety of anesthetic services to labor units nationally, observational data on workforce, workload, and clinical outcomes should be collected prospectively in all labor units in Israel.

**Supplementary Information:**

The online version contains supplementary material available at 10.1186/s13584-021-00460-2.

## Background

Israel is in the midst of an anesthesia workforce deficit [1]. In these circumstances, obstetric anesthesia services may be perceived as neither life-saving nor profitable, and may suffer disproportionate shortages of workforce allocation [2]. In a nationwide survey of all maternity units in Israel performed in 2005 [2], we found that the provision of anesthesia workforce supply to maternity units is often inadequate to meet workload demands and that hospitals with no anesthesiologist dedicated to the maternity unit had longer epidural waiting times, lower rates of epidural analgesia and higher rates of general anesthesia for emergency CS (associated with increased maternal and neonatal risk).

In the current study, we aimed to conduct an objective observational snapshot study of the obstetric anesthesia service in Israel’s maternity units in order to assess the effect of the workforce supply to workload demand ratio on outcomes of obstetric care that will affect patient satisfaction and patient safety. We hypothesize that as the ratio of workforce supply to workload demand decreases, that patient satisfaction and patient safety outcomes will deteriorate.

There has been both a reduction in anesthesia workforce supply and an increase in anesthesia workload demand over recent years in Israel [1]. Reduced anesthesia workforce supply is related to the unpopularity of anesthesia as a specialty among Israeli medical school graduates (less than 1% of Israeli graduates chose the specialty at the time of that study [1]) and to the fact that the ranks of the subspecialty were traditionally filled by immigrant physicians. As immigration to Israel declined, there has been reduced recruitment of young anesthesiologists, with the result that the population of anesthesiologists is both dwindling and aging [1]. Increased anesthesia workload demand is due in part to an increase in the number and complexity of surgeries performed (due in turn to an increase in population size, and an increase in surgical procedures performed per capita, particularly in the disproportionately increasing elderly population). Furthermore, anesthesia workload demand increased with the growing need for anesthesia services outside of the operating room (including intensive care units, acute and chronic pain services and sedation teams). In particular, Weissman et al highlighted a marked increase in the demand for obstetric analgesia and anesthesia [1]. The study identified an increased demand for obstetric anesthesia services due to both an increase in the requests for labor analgesia and a marked increase in the cesarean delivery rate (rising from 9.6% in 1992, [3] to 17–18% by 2004 [4]). The increased demand for obstetric anesthesia services was predicted to grow disproportionately [1, 2, 5]. This has leveled at around 20%, nationwide in the last decade [2, 5]. Although the C-section rate has leveled off over the past few years, the number of births have increased (from 136,500 in 2000 to 183,400 in 2017),^1^ so that the absolute number of C-sections has increased, so increasing the burden upon anesthesiologists.

Several nationwide surveys have been performed over the past 10 years in which all obstetric anesthesia unit directors in Israel participated [2, 5, 6]. These surveys identified a wide variation in the relationship between obstetric anesthesia workforce supply and workload demands in maternity units. Of the 25 hospitals surveyed in the 2009 survey, only 11 had an anesthesiologist assigned to the labor ward on a 24 h-7 day basis (24/7) [2]. Although all hospitals with more than 7500 deliveries had dedicated 24/7 anesthesia coverage, a 24/7 service was only available in 3 out of 8 hospitals with 5000–7500 deliveries. In the absence of a dedicated anesthesiologist for the labor ward, both routine and emergent obstetric anesthesia care has to compete with emergency surgery in the operating rooms. In that study, hospitals with a dedicated anesthesiologist in the labor ward 24 h a day had almost a two-fold increase in epidural analgesia rate and half the epidural waiting time compared to hospitals where the service was based on calling an anesthesiologist from the main operating rooms. Furthermore, there was an inverse relationship between epidural administration rate in labor and the choice of general anesthesia for emergency cesarean delivery. A follow-up study, published by Shatalin, et al. in 2019 [5], showed an improvement in the number of hospitals with dedicated obstetric anesthesia staffing (from 44% of hospitals to 76% of them). This increase in workforce allocation was driven in part by an increase in obstetric anesthesia workload, with epidural administration increasing from 50 to 60% of all labors nationally [5].

While these data are important, it must be remembered that these were based on surveys of anesthesiology directors rather than on observational data, and that workload-demand: workforce-supply relationships were not assessed, hence the need for the current observational study.

It has been long recognized that apart from the provision of adequate pain relief on request, that epidural analgesia in labor has also made an important contribution to the safety of mothers and babies during labor. This is because a functioning epidural catheter can be rapidly used for the provision of epidural anesthesia for urgent cesarean delivery avoiding the need for emergent general anesthesia [7]. General anesthesia is associated with a 10-fold higher risk of airway complications among women undergoing cesarean delivery than among non-obstetric patients [8]. Maternal death due to anesthesia is the sixth leading cause of pregnancy-related death in the United States [8, 9]. Most anesthesia-related deaths occur during general anesthesia for cesarean delivery. The risk of maternal death from complications of general anesthesia is 17 fold higher than that associated with regional anesthesia [10]. Hence regional anesthesia is the standard of care for cesarean delivery [7]. Recognition of the risks to the mother associated with general anesthesia has led to an increased use of spinal and epidural anesthesia for both elective, urgent and most emergency cesarean deliveries [9]. This practice change may be related to the decrease in anesthesia-associated maternal mortality from 4.3 to 1.7 per 1 million live births in the United States [11]. Part of the accepted role of the obstetric anesthesiologist is to identify important risk factors for anesthesia (such as physical signs predicting a difficult intubation, a diagnosis of pre-eclampsia and concomitant morbid obesity), and so pre-emptively offer epidural analgesia early in the course of labor [7]. This is particularly important where there are signs of impending urgent or emergent cesarean delivery (such as poor progress in labor, with variable decelerations in fetal heart rate). This degree of proactive anticipation can only occur if the anesthesiologist is present in the labor ward and is not just called when an epidural is requested.

It should be stressed that the questionnaire-based surveys [2, 5] above were not based on collected observational data and so was not able to assess *why* general anesthesia was more commonly used for emergency cesarean delivery in hospitals with limited anesthesia cover. We hypothesize that the lack of an available anesthesiologist to proactively place an epidural catheter during labor or to administer a supplemental dose of epidural local anesthetics in time to convert epidural labor analgesia to epidural surgical anesthesia was an important contributing factor. However, only prospective observational data of the sort collected in our current study can provide that information. Furthermore, the Weiniger et al survey [2] was unable to assess the numbers and proportions of women who did not receive epidural analgesia for labor despite requesting it, nor how many had to wait for long periods in pain until an anesthesiologist eventually became available.

## Methods

### Aims

This study aimed to conduct a prospective, observational, snapshot of the obstetric anesthesia service in all 27 maternity units in Israel, in order to assess the ratio between the anesthesia workload demand and the anesthesia workforce supply in different centers and then to assess the impact of this workload/workforce ratio (WL/WF) on outcomes of peripartum anesthesia care that affect quality and safety. We hypothesized that as the WL/WF ratio increases, that quality and safety outcomes will deteriorate.

We also aimed to investigate differences in the quality and safety of care among women giving birth in hospitals with different populations (serving predominantly Jewish Orthodox, secular or Arab populations) and different geographical locations (periphery versus central). We also assessed the relation between timing of the request for epidural analgesia (day versus night, weekend versus weekdays) and the epidural waiting time.

### Design and setting

In this prospective observational snapshot study, trained observers attended Israeli maternity units for a 72-h observation period, to collect data based on observation of clinical practice, patient care, and review of records. Out of all 27 labor wards in Israel, only 12 were eventually included in the study. Seven large medical centers belonging to “Clalit” HMO had to be excluded, after the Clalit HMO refused to allow their hospitals to participate, in some cases after IRB approval had been obtained locally. The other eight hospitals excluded had administrative difficulties obtaining local IRB approval. Observers were medical students, who were trained by the researchers on how to fill observational sheets. Observation periods were divided to 9 consecutive shifts of 8 h duration. First shift always began on Wednesday at 08:00 AM and the last shift ended on Saturday at 08:00 AM. We thus managed to collect data from both day and night shifts as well as mid-week and weekend.

On every shift, in every labor unit, there was one observer who was located in the nurses’ station, from where the activity in all labor rooms was monitored. Although the observers were not permitted to access the labor rooms, they were able to ask direct questions of the midwife.

Data regarding timing were collected: time of midwife requesting an anesthesiologist to perform an epidural, and the time of anesthesiologist arrival. Information regarding the reasons why women gave birth without the use of epidural analgesia was also gathered, in order to differentiate those who delivered without epidural due to delay in arrival of the anesthesiologist, from other reasons such as patient choice or medical contraindications. Other variables that were collected by interviewing the head midwife were: number of anesthesiologists dedicated to covering the labor ward, reasons for anesthesiologist unavailability, and reasons for the use of general anesthesia in urgent or emergent cesarean delivery. Data regarding number of overall deliveries per year, rates of cesarean sections and rates of epidural analgesia administrations in each hospital were also gathered. All data from observation sheets were transferred to SPSS software (see below) for analysis.

#### Reliability

To test the reliability of the prospectively collected observational data, a comparison was conducted by hand between data from the observational sheets and the written labor ward record for deliveries and for epidurals in a sample of 102 deliveries (17.7% of the total study population). The comparison included the following variables: epidural analgesia administered (yes/no), time of epidural and time of delivery. In two cases (2%), the time of epidural analgesia administration was inconsistent with epidural ledger; hence the agreement (‘Kappa’) index was equal to 0.97 (*p* < 0.0001).

#### Sample size calculation

As this was a national ‘snapshot’ study, full participation was assumed from all 27 obstetric units in Israel. With a national annual rate of 125,000 births [2], a 72-h sample would attain ~ 1025 cases to analyze. Based on a national median of 50% epidural analgesia [2], this calculated sample size (1025) should have allowed us to differentiate epidural analgesia administration rates between 30 and 70% with a 95% confidence interval of 2% (±1%), even after stratifying the hospitals by different demographic subtypes. This sample size should also have allowed us to differentiate a 2% incidence of Cesarean sections under general anesthesia with a 95% confidence interval of 1.3–3%. Eventually, seven large hospitals were excluded due to the refusal of Clalit HMO to allow their hospitals participate in this study, and eight others due to lack of IRB approval. The remaining 12 hospitals supplied us with 513 cases (50% of the predicted sample size); these comprised both regular vaginal deliveries and urgent and emergent cesarean deliveries. This directly affected the statistical significance and statistical power of the inferences sought. Accordingly, we made the decision to limit analysis to descriptive statistics rather than inferential statistics.

### Independent variables

1. Obstetric anesthesia workload (WL) demand – including number/year of deliveries with epidural analgesia and number/year of cesareans deliveries. In order to receive a single number for each hospital, these two variables were merged into one: Obstetric Anesthesia Activity Index (OAAI). This has been described previously [7]. The OAAI is quantified in hours/day units. It is calculated using the following equation:
  $$ OAAI=\frac{\left( no. of- epidurals- per- yr\times 0.75\right)+\left( no. of- cesareans- per- yr\times 1.5\right)}{365} $$

The equation takes into consideration a typical ratio of 2:1 regarding time (in hours) spent on anesthesia for a cesarean delivery compared with the time required to perform labor epidural analgesia, divided by an average of 365 days per year [7].

2. Obstetric anesthesia workforce (WF) supply – the number of anesthesiologists dedicated specifically to the delivery unit, in every shift (morning, evening and night, during the week and during weekends).

OAAI was divided by the workforce variable in order to obtain a single number which represents the workload / workforce ratio (WL/WF) for each hospital.

### Dependent variables

#### Quality of care

1. Epidural response time; the elapsed time between the midwife calling the anesthesiologist until the arrival of the anesthesiologist.
2. Amount of women who gave birth without epidural analgesia, even though they requested it. This variable only includes cases in which the reason for delivering without epidural analgesia was due to delay in the arrival of the anesthesiologist.

#### Safety of care

Based on data that general anesthesia is associated with higher risk than regional anesthesia in cesarean delivery, especially urgent surgery (see introduction), we determined that general anesthesia in urgent cesarean delivery performed because of the late arrival of the anesthesiologist was a good safety outcome measure. We examined all urgent cesarean deliveries; when performed under general anesthesia we identified the predominant reason for the choice of anesthesia and identified those where this was predominantly due to the late arrival of the anesthesiologist.

### Statistical analysis

We used SPSS 19 for Windows (IBM Corp, Armonk, NY) software. Descriptive statistics were used to describe the rates of vaginal and cesarean delivery, rates of epidural analgesia, rates of general anesthesia for cesarean delivery, epidural waiting times and workforce supply data.

## Results

### Sample characteristics

Twelve Israeli hospitals (7 governmental and 5 private) participated in the study: Shamir-Asaf HaRofe (Rishon Lezion), Poria (Tiberias), Mayanei Hayeshua (Bnei Brak), Ichilov (Tel Aviv), Wolfson (Holon), Barzilai (Ashkelon), Bnei Zion (Haifa), Hadassah Ein Karem (Jerusalem), Bikur Cholim (Jerusalem) and Shaarei Zedek (Jerusalem). Excluded hospitals included the seven hospitals of Clalit HMO: Rabin-Beilinson (Petach Tikva), Yoseftal (Eilat), Soroka (Beersheva), HaEmek (Afula), Meir (Kfar Saba), Kaplan (Rehovot), Carmel (Haifa). Also excluded were hospitals that did not obtain IRB approval: the Nazareth hospitals (English, Italian and French), Sheba (Tel Hashomer), Laniado (Netanya), Hillel Yaffe (Hadera), Rambam (Haifa).

Three hospitals were defined as located in Israel’s periphery (the remainder center); three hospitals were defined as serving a predominantly Jewish Orthodox population. Obstetric anesthesia workload demand varied widely between hospitals, with an OAAI ranging from 4.59 to 25.11. During 72 continuous hours of observation in 12 hospitals, we gathered data on 575 deliveries. Of these, 513 (89.2%) were vaginal deliveries and 62 (10.8%) were unscheduled cesarean deliveries. Elective cesareans were not included in the study. Three hundred forty (59.1%) women requested epidural analgesia; of these, 321 women received their requested epidural. This includes women who received epidural for vaginal delivery but eventually underwent cesarean delivery. Two hundred sixteen deliveries (37.6%) took place at hospitals defined as serving a predominantly Jewish Orthodox population; out of these, 197 (91.2%) were regular vaginal deliveries and 19 were cesarean deliveries. Eighty two deliveries (14.3%) took place in peripheral hospitals; of these 72 (87.8%) had vaginal deliveries and 43 (52.4%) requested epidural analgesia during labor. The rate of request for epidural analgesia was similar to that among women from the majority Orthodox population hospitals – 113 (52.3%). In comparison, secular centers had 227 requests for epidurals (63.2%) and central hospitals received 297 requests (60.2%) (Table 1).

**Table 1:** Reported annual workforce and workload variables in the various medical centers

Center	Workforce (WF) variable				Weekly Anesthesia Staff Needed^a^	Workload (OAAI)	OAAI/WF
	Weekday Shift			Weekend Shift			
	Morning	Evening	Night	Morning/Evening/Night			
1	2	1	1	1	26	12.240	0.47
2	0	0	0	0	0	8.780	–
3	2	1	1	0	20	21.130	1.06
4	1	2	2	2	37	25.110	0.68
5	1	1	1	2	27	9.430	0.35
6	2	2	2	2	42	14.900	0.35
7	1	0	1	0	10	6.900	0.69
8	1	1	1	1	21	8.310	0.39
9	2	2	2	2	42	15.700	0.37
10	0	0	0	0	0	4.590	–
11	2	2	1	2	37	6.020	0.16
12	1	1	1	1	21	4.830	0.23

^a^ Calculated from the preceding columns for 5 weekdays and 2 weekend days (e.g. for Center 1 the calculation is [5 × (2 + 1 + 1) + 2 × (1 + 1 + 1)] = 26)

The number of anesthesiologists providing dedicated cover only to the labor ward ranged from 0 to 2 in different hospitals and on different shifts. Two hospitals did not have a dedicated anesthesiologist for the labor ward at any shift, day or night. Two other hospitals allocated 2 anesthesiologists exclusively to the labor ward on a regular basis. The annual reported number of deliveries, cesareans and epidurals, together with the OAAI for each hospital are presented in Table 2. The number of deliveries, unscheduled cesareans and epidurals (during the 72 h of observation) are presented in Table 3.

**Table 2:** Annual delivery, Cesarean section and epidural rates in the various medical centers during the study

Center	Annual data			
	N (%)			
	Deliveries	CS (out of all deliveries)	Epidural	OAAI
1	6068	1310 (21.6)	3337 (55.0)	12.240
2	4823	871 (18.1)	2531 (52.5)	8.780
3	14,754	1777 (12.0)	6734 (45.6)	21.130
4	11,400	2587 (22.7)	7051 (61.9)	25.110
5	4834	1136 (23.5)	2320 (48.0)	9.430
6	8184	1644 (20.1)	3965 (48.4)	14.900
7	3546	919 (25.9)	1520 (42.9)	6.900
8	4116	828 (20.1)	2392 (58.1)	8.310
9	10,309	1092 (10.6)	5458 (52.9)	15.700
10	2628	446 (17.0)	1343 (51.1)	4.590
11	5708	440 (7.7)	2054 (36.0)	6.020
12	3348	692 (20.7)	968 (28.9)	4.830
Total	79,718	13,742 (17.2)	39,673 (49.8)	11.495

(Note - this table includes all annual data, including elective CS. Therefore, the denominator in the epidural rate in this table is calculated as the total number of deliveries, which includes elective CS)

**Table 3:** Number of deliveries, unscheduled cesareans and epidurals (during the 72 hours of observation)

Center	Data from 72 hours of observation				
	N (%)				
	Regular Vaginal Deliveries	CS	Asked for Epidural	Delivered with Epidural	Epidural Response Rate
1	40 (87.0)	6 (13.0)	29 (63.1)	29 (63.1)	100.0
2	26 (83.9)	5 (16.1)	15 (48.4)	12 (38.7)	80.0
3	88 (86.3)	14 (13.7)	54 (53.5)	55 (54.5)	101.9
4	57 (83.8)	11 (16.2)	49 (72.1)	48 (70.6)	98.0
5	37 (84.1)	7 (15.9)	31 (70.5)	31 (70.5)	100.0
6	68 (95.8)	3 (4.2)	43 (60.6)	41 (57.7)	95.3
7	16 (94.1)	1 (5.9)	17 (100.0)	15 (88.2)	88.2
8	21 (87.5)	3 (12.5)	17 (70.8)	14 (58.3)	82.4
9	65 (97.0)	2 (3.0)	39 (58.2)	36 (53.7)	92.3
10	19 (90.5)	2 (9.5)	10 (47.6)	9 (42.9)	90.0
11	44 (93.6)	3 (6.4)	20 (42.6)	15 (31.9)	75.0
12	32 (86.5)	5 (13.5)	16 (43.2)	16 (43.2)	100.0
Total	513 (89.2)	62 (10.8)	340 (59.1)	321 (55.8)	94.4

(Note: The epidural rate calculation’s denominator is the total number of deliveries (including unscheduled CS). As explained in methods, elective CS were not included in the 72 hours observation)

The characteristics of women who did not receive epidural analgesia are summarized in Table 4. Eighteen women (3.1% of all deliveries) requested an epidural but did not receive one, due to the delayed arrival of the anesthesiologist. This number was only a small proportion of the women who requested epidural analgesia but did not receive it for other reasons; 55 women (9.6% of all deliveries) requested an epidural but labor was so advanced that there was no time for the midwife to call the anesthesiologist. Seven women (1.2% of all deliveries) had a contraindication to epidural analgesia and 133 (23.1% of all deliveries) did not receive an epidural by choice.

**Table 4:** Reasons for deliveries without epidural analgesia

Center	Patient Refusal^a^	Late arrival of anesthesiologist	Epidural not performed due to rapid labor progress^b^	Contra-indication to epidural
1	10 (83.3)	1 (8.3)	1 (8.3)	0
2	11 (68.8)	2 (12.5)	2 (12.5)	1 (6.3)
3	24 (60.0)	2 (5.0)	12 (30.0)	2 (5.0)
4	6 (37.5)	2 (12.5)	6 (37.5)	2 (12.5)
5	5 (45.5)	0	5 (45.5)	1 (9.1)
6	19 (65.5)	2 (6.9)	8 (27.6)	0
7	0	2 (100.0)	0	0
8	4 (44.4)	2 (22.2)	2 (22.2)	1 (11.1)
9	23 (79.3)	3 (10.3)	3 (10.3)	0
10	6 (54.5)	1 (9.1)	4 (36.4)	0
11	15 (60.0)	1 (4.0)	9 (36.0)	0
12	10 (76.9)	0	3 (23.1)	0
Total	133 (62.4)	18 (8.5)	55 (25.8)	7 (3.3)

^a^ Patient offered an epidural but declined

^b^ Patient was interested in an epidural but labor was so fast that there was not time for the midwife to request it; alternatively, the time from epidural request to crowning prior to delivery was so short that the arrival of the anesthesiologist was not the limiting factor, but rather the very rapid progress of labor

## Discussion

Our study addressed the hypothesis that a shortage of anesthesiologists in Israel will impair the *quality* and *safety* of peripartum anesthesia care.

The *quality* was primarily assessed by measuring the epidural waiting time, from patient request for an epidural until the arrival of the anesthesiologist; in addition we recorded the number of deliveries where epidural analgesia was not provided at all due to the lack of availability of the anesthesiologist, or his late arrival. The *safety* component of peripartum anesthesia care was assessed by the number of general anesthetics administered for urgent cesarean delivery in cases where there was no contraindication to regional anesthesia.

There was large variability between the different medical centers in respect to epidural waiting times. In most cases they were not normally distributed and extreme outliers were observed, accordingly central tendency was presented as median rather than mean. Median waiting times ranged from 5 to 28 min in the different hospitals. Anesthesia workforce varied between 0 to 2 anesthesiologists dedicated to the delivery unit. To standardize the anesthesia workload in relation to anesthesia workforce, we used the OAAI to calculate the WL/WF ratio.

Comparison of median epidural waiting times between different shifts, geographical location (central versus peripheral) and demographics (population served) suggested that the median epidural waiting time was shorter in hospitals serving a predominantly Orthodox Jewish population (6 min), nearly half the median epidural waiting time observed in hospitals serving a general or secular population (Table 5).

**Table 5:** Observed Anesthesiologist arrival times, split by demographic and temporal characteristics

Center/Shift Characteristics	Delay in receiving Epidural Analgesia			Analgesia never received	Total^a^	***P***-value
	< 30 min	30–60 min	> 60 min			
Peripheral Hospitals	33 (78.6)	7 (16.7)	1 (2.4)	1 (2.4)	42	0.582
vs.						
Central Hospitals	223 (76.4)	37 (12.7)	21 (7.2)	11 (3.8)	292	
Majority Jewish Orthodox	99 (90.8)	5 (4.6)	3 (2.8)	2 (1.8)	109	***< 0.0001***
vs.						
Majority Secular	157 (69.8)	39 (17.3)	19 (8.4)	10 (4.4)	225	
Daytime Shift	180 (75.9)	31 (13.1)	18 (7.6)	8 (3.4)	237	0.700
vs.						
Evening/Night Shift	76 (78.4)	13 (13.4)	4 (4.1)	4 (4.1)	97	
Weekday Shift	178 (74.5)	36 (15.1)	17 (7.1)	8 (3.3)	239	0.350
vs.						
Weekend Shift	78 (82.1)	8 (8.4)	5 (5.3)	4 (4.2)	95	

^a^6-data points missing for date and time

Only 2 out of 12 medical centers allocated fewer anesthesiologists to the labor ward during the weekend, compared to a regular weekday. Similarly, only 3 out of 12 medical centers allocated fewer anesthesiologists to the labor ward afterhours than during the work day. This may explain in part why there was no significant difference in median epidural waiting times between the weekdays and weekends. In addition, the median epidural waiting time in peripheral hospitals were similar to those in the larger central hospitals. The workload/workforce relationship (OAAI/WF) may be difficult to interpret in hospitals with very low workload. For example, in Table 1, the WF was low or anesthesiologists were only available on request from the operating room, without any increase in the epidural waiting time, suggesting that this workforce provision was adequate to meet the service workload.

The *safety* outcome was the use of general anesthesia for unscheduled cesarean delivery. Only 10.8% of all deliveries in this sample were by cesarean delivery; while this is far lower than the annual cesarean delivery rate of 17.2% in these hospitals, this number does not include the elective cesareans that were excluded from this sample. Only 3 patients in our entire sample underwent general anesthesia due to delayed arrival of the anesthesiologist (Table 6). Similar findings have been recently exhibited in Israeli studies on peripartum anesthesia care [5, 6].

**Table 6:** Observed frequency of Cesarean Section under general anesthesia and their causes

Center	Cesarean Sections [N (rate)]	% General Anesthesia	Reasons for General Anesthesia		
			Maternal	Late arrival of anesthesiologist	Epidural malfunction
1	6 (13.0)	50.0	1 (33.3)	2 (66.7)	0
2	5 (16.1)	0.0			
3	14 (13.7)	21.4	2 (66.7)	0	1 (33.3)
4	11 (16.2)	0.0			
5	7 (15.9)	14.3	1 (100.0)	0	0
6	3 (4.2)	33.3	1 (100.0)	0	0
7	1 (5.9)	0.0			
8	3 (12.5)	66.7	0	1 (50.0)	1 (50.0)
9	2 (3.0)	50.0	?		
10	2 (9.5)	0.0			
11	3 (6.4)	?			
12	5 (13.5)	40.0	1 (50.0)	0	1 (50.0)
Total	62 (10.8)	13 (21.0)	6 (46.2)	3 (23.1)	3 (23.1)

These definitions of quality and safety have been used by obstetric anesthesiologists in the United States since 1981, re-iterated in 1997 and 2005 [12, 13], and used as benchmarks for studies parallel (and similar) to ours in California [14], Georgia [15] and Croatia [16]. A 30 min time for arrival of the anesthesiologist as a maximum acceptable delay from being called for an epidural is clearly stated as a strong recommendation in the 2020 Royal College of Anaesthetists Guidelines for the Provision of Anaesthesia Services for an Obstetric Population.^2^ That guideline adds: “only in exceptional circumstances should this period be longer, and in all cases attendance should be within one hour. This should be the subject of regular audits”.

There is no set benchmark for the percentage of deliveries that should meet this 30-min deadline. Each service should aim to meet this minimum standard in all patients. Within this 30 min limit, clearly the faster an anesthesiologist can arrive the better. Any service that offers critical, time-dependent, urgent care certainly should not aim for providing staff to meet an “average” day, because on a busy day the service will be overwhelmed leading to sub-standard care with potentially severe adverse events. Some level of inbuilt redundancy is needed for safety and quality. There are two approaches to providing this staffing resource:

- *Pro-active approach:* The workforce supply should be designed to meet the needs of a busy day. On an “average” day, the extra staff are used to improve the service to include other important tasks which may not currently get prioritized because of poor staffing numbers. These tasks are generally not time-dependent and can be interrupted for epidural analgesia or CS anesthesia. Examples include ante-natal visits, anticipatory labor rounds to identify high-risk patients who should be offered epidurals early in labor (e.g. poor progress in labor with non-reassuring fetal heart rate), post-natal visits to identify complications, audits, quality assurance, and resident teaching. This pro-active staffing approach improves the service and is preferred, but it may not be possible in the presence of cash-strapped institutions or if there is an overall staffing shortage.
- *Reactive approach:* The workforce supply is designed to meet the needs of an “average” day’s workload. Back-up is provided from the anesthesia department which commits to provide the extra staff immediately from other services if needed (e.g. bringing an additional anesthesiologist from the main operating room). This approach maintains the epidural service as a reactive service but does not provide the other improvements in service offered by the pro-active approach. It also does not provide any guarantees that this extra *reactive* workforce supply would be available if a busy day in the labor ward coincided with a busy day in the main operating room.

There are myriad priorities related to the provision of health care funding and resources; these vary between countries and between institutions. Epidural analgesia for labor and anesthesia for cesarean delivery are not new priorities on any national scale that makes them eligible for special national funding. This is in stark contrast to new services with costly infrastructures, such as stroke centers (with expensive invasive neuroradiology suites) or new ECMO service centers.

Israel has a universal healthcare system where most resources are allocated on a centralized basis, and there is no separate billing for anesthesia services. However, in the twenty-first century, all women in high income countries understand that they have rights, which include the provision of painless labor if they want it, and safe cesarean delivery if they should need it. The provision of these rights is a standard expectation of modern childbirth.

In addition to rights, in a competitive market with free flow of information, patients understand that they have choice. The comparative length of time waiting for an epidural, particularly at night, is a frequent topic in internet chat rooms. Additionally, the labor ward is a storefront window through which young families view the entire medical center, not just the obstetric service.

This is one of the motivations for the Israel Association of Obstetric Anesthesia, the Israel Society of Maternal-Fetal Medicine and the Israel Association of Midwives to co-operate in the building of this research program, and provide this information to hospital directors. These factors motivate hospitals to dedicate resources for this service and enable it to function in a sufficient level; no hospital director wants to find itself placed at the bottom of the national or regional tables.

The reader may judge if this was one of the motivations for Clalit HMO to refuse to allow their hospitals to join in the project. Ultimately, each hospital decides on the human resources it will dedicate to obstetric anesthesia services. Increases in allocation have hitherto been typically based on increased demand or the response to complaints from patients, anesthesiologists, obstetricians and midwives. In this study we describe a study methodology for a three-day national snapshot of all Israel’s maternity units, and aimed to provide some objective data upon which to base an objective workforce requirement based on measurable levels of workload.

### Study limitations

There are several limitations to this study. Firstly, there were considerably fewer deliveries assessed than expected. This was a direct consequence of the refusal by Clalit HMO to allow their hospitals to participate in the study and the difficulties that other hospitals found in obtaining IRB approval for this observational assessment. The lower sample size impacted the power of the study and forced us to perform only descriptive rather than inferential statistics.

Additionally, this introduced an element of selection bias into the hospitals that participated in the study. The refusal of a single large HMO to allow its hospitals to participate in this study may be regarded as non-participation bias. We cannot speculate as to whether this was based on any subjective concern that the WL/WF ratio in their hospitals, or the quality and safety outcomes may not reflect the national average. Similarly, six of the eight hospitals excluded due to administrative difficulties obtaining local IRB approval were small, low workload hospitals (such as all the hospitals in Nazareth) and their exclusion is a sectorial bias.

There was some degree of geographical bias. The central region is represented reasonably appropriately; Shamir-Asaf HaRofe (Rishon Lezion), Mayanei Hayeshua (Bnei Brak), Ichilov (Tel Aviv), and Wolfson (Holon) are included, while Rabin-Beilinson (Petach Tikva), Meir (Kfar Saba), Sheba (Tel Hashomer), Laniado (Netanya) are not. However outside of the central region, there is geographical bias, with over-representation of Jerusalem and under-representation of the north and south. Clalit have no hospitals in the Jerusalem area, the highest birthrate area in the country, and so all the Jerusalem hospitals (Bikur Cholim, Hadassah Ein Karem and Hadassah Mt Scopus) were included. By comparison, in the north only Poria (Tiberias), Ziv (Tzfat), and Bnei Zion (Haifa) were included, while HaEmek (Afula), Carmel (Haifa), the Nazareth hospitals, Galilee Medical Center (Nahariya), Hillel Yaffe (Hadera), and Rambam (Haifa) were not. In the south only Barzilai (Ashkelon) was included, while Yoseftal (Eilat), Soroka (Beersheva), and Kaplan (Rehovot) were not.

We attempted to compare the characteristics (e.g. annual delivery numbers, cesarean rates, epidural rates, percentage of nulliparous and grand-multiparous labor, and population characteristics) of the hospitals remaining in the study with those that were excluded. Data obtained from the Israel Ministry of Health and from the Israel Society of Maternal Fetal Medicine were blinded by hospital. We analyzed data from national surveys of obstetric anesthesia units collected in 2007 [7] and 2018 [5], (see supplementary Table 1). Based on this data, the hospitals included in the OASIS study delivered 48% of all the deliveries nationally. There was a slightly lower cesarean delivery rate in the hospitals included in the study than in hospitals excluded, possibly reflecting the well-described lower cesarean delivery rate in hospitals serving predominantly Hareidi orthodox Jewish communities.

An example of this variation in seen in Table 3; the epidural rates ranged from 32 to 88%. Israel is a country with a rich mosaic of different cultural norms. These are reflected in a remarkably high range of epidural rates between different hospitals, serving different local populations. This is a phenomenon that has been published before, both by our group [2, 5, 7] and by others [17].

We did not distinguish in this study between attending anesthesiologists and resident anesthesiologists, or between junior and senior residents. In the US, billing requirements generally dictate that all epidurals or cesareans are provided directly or are supervised, by an attending anesthesiologist (or a nurse anesthetist in some states); residents and fellows generally need to be directly supervised. In Israel, billing requirements are very different and most labor wards are only staffed by residents at night and weekends. In a third of Israel’s hospitals, new residents may serve in delivery rooms even within the first six months of their residency program without any direct supervision of an attending anesthesiologist [5]. In another third, they only start unsupervised obstetric anesthesia after a minimum of six months of training, and in a third they are only allowed to provide unsupervised obstetric anesthesia much later in their training [5].

Observers were not allowed to enter the labor rooms, as this would have required obtaining personal written consent from every parturient. Apart from the logistic problems inherent in obtaining consent on such a scale, the women who would inevitably refuse would also cause selection bias. As a consequence, the data was recorded from observers in the nursing station. They asked midwives for data, such as time of epidural request, and anesthesiologists’ data, such as reason for general anesthesia. In many cases this was not difficult as calls to the anesthesiologist were usually made from the nurse’s station. Also, although observers were centrally trained by the same researcher, their observations of time measurement may not have been applied identically and this may have been a source of inter-observer bias. While both these factors may have been potential sources of error, they are unlikely to have been a differential bias, with both overestimations and underestimations. Similarly, since observers were dependent on the midwives for data, there may have been an element of response bias in the willingness of midwives to cooperate. Heavy workload shifts in the labor unit may have been expected to decrease cooperation and may have impacted the accuracy of data; but again this is likely to be a non-differential bias. Finally, there may be a lead-time bias in this study. Contrary to the initial study plan, where all labor units would be assessed in parallel, units were assessed sequentially over a period of 18 months. This was due entirely to the difficulties involved in attempting to obtain IRB approval in 27 medical centers. During such a long sampling period national trends in anesthesia workforce and obstetric workload may have occurred.

We excluded elective CS from this sample. It is an accepted principle for the organization of obstetric anesthesia services that the staffing ratios should separate elective surgery from unscheduled anesthetic services such as labor epidural or urgent cesarean section. This is stated in the 2020 Royal College of Anaesthetists Guidelines for the Provision of Anaesthesia Services for an Obstetric Population^3^: “There should be a duty anaesthetist immediately available for the obstetric unit 24/7. This person’s focus is the provision of care to women in labour or who, in the antenatal or postpartum period, require medical or surgical attention. The role should not include undertaking elective work during the duty period.” Combining the responsibilities of anesthesia providers to include both elective surgery and unscheduled epidurals and urgent CS makes it likely that anesthesiologists will be less available for women in labor, and also impairs the timing of elective cases. Elective CS should be scheduled for dedicated operating lists at a particular time and should have surgery as planned at that time, in exactly the same way as occurs for scheduled non-obstetric surgeries, which are not expected to have to compete with trauma and other urgent operations.

## Conclusions

This study aimed to provide objective data to assess the hypothesis that the ability to provide quality and safe obstetric anesthesia services requires adequate workforce allocations. Furthermore, we were hoping that this data would be able to provide a threshold or minimum acceptable workload/workforce ratio. We did not observe data to substantiate this hypothesis, or to provide this yardstick. We do show that the OAAI is a better predictor of WL than delivery numbers alone, especially concerning WF shortage. We accordingly believe that it would be a dangerous and simplistic misinterpretation to infer from this study that the quality and safety of obstetric anesthesia services is independent of the workload/workforce ratio. Ideally the Israeli Health Ministry, as health system regulator, would initiate a large study of all labor units, without the option for individual hospitals to refuse to participate. Until such a time, it is reasonable to suppose that hospitals with both smaller workforce allocations and heavier workload demands will have an inferior service. Ultimately, consumer power, the word of mouth of unsatisfied laboring women will provide the market force to initiate change.

## Supplementary Information

**Additional file 1: Supplementary Table 1.** Characteristics of hospitals that participated and that did not participate in the OASIS study. Data from Weiniger [2] and Shatalin [5] for years 2007 and 2018 respectively.

## Data Availability

Not applicable.

## Abbreviations

CS: Cesarean section(s)
HMO: Health maintenance organization
IRB: Institutional review board
NIHP: National Institute for Health Policy Research
OAAI: Obstetric Anesthesia Activity Index
OASIS: Obstetric anesthesia services in Israel snapshot
WF: Work force
WL: Workload

## Footnotes

1 https://www.health.gov.il/publicationsfiles/birth_2000_2017.pdf accessed on 22/11/2020

2 https://www.rcoa.ac.uk/gpas/chapter-9 accessed on 7/6/2020

3 https://www.rcoa.ac.uk/gpas/chapter-9 accessed on 7/6/2020

## References

[1] Weissman C, Eidelman LA, Pizov R, Matot I, Klein N, Cohn R (2006). The Israeli anesthesiology physician workforce. Isr Med Assoc J.

[2] Weiniger CF, Ivri S, Ioscovitch A, Grimberg L, Evron S, Ginosar Y (2010). Obstetric anesthesia units in Israel: a National Questionnaire-Based Survey. Int J Obstetric Anesthesia.

[3] Mor-Yosef S, Samueloff A, Schenker JG (1992). The Israel perinatal census. Asia Oceania J Obstet Gynaecol.

[4] Cohain JS. Midwifery in Israel. Midwifery Today Int Midwife. 2004 Autumn;(71):50–1. PMID: 15536943. 15536943

[5] Shatalin D, Weiniger CF, Buchman I, Ginosar Y, Orbach-Zinger S, Ioscovich A (2019). A 10-year update: national survey questionnaire of obstetric anesthesia units in Israel. Int J Obstet Anesth.

[6] Orbach-Zinger S, Ioscovich A, Aviram A (2014). National survey of postoperative pain control after cesarean delivery. Isr Med Assoc J.

[7] Ginosar Y, Ioscovich A, Weissman C, Calderon-Margalit R, Weiniger CF (2012). Comparison of the obstetric anesthesia activity index with total delivery numbers as a single denominator of workload demand in Israeli maternity units. Isr J Health Policy Res.

[8] Berg CJ, Atrash HK, Koonin LM, Tucker M (1996). Pregnancy-related mortality in the United States, 1987-1990. Obstet Gynecol.

[9] Hawkins JL, Koonin LM, Palmer SK, Gibbs CP (1997). Anesthesia-related deaths during obstetric delivery in the United States, 1979-1990. Anesthesiology.

[10] Khor LJ, Jeskins G, Cooper GM, Paterson-Brown S (2000). National obstetric anaesthetic practice in the UK 1997/1998. Anaesthesia.

[11] Eltzschig HK, Lieberman ES, Camann WR (2003). Regional anesthesia and analgesia for labor and delivery. N Engl J Med.

[12] Hawkins JL, Gibbs CP, Orleans M, Martin-Salvaj G, Beaty B (1997). Obstetric anesthesia work force survey, 1981 versus 1992. Anesthesiology..

[13] Bucklin BA, Hawkins JL, Anderson JR, Ullrich FA (2005). Obstetric anesthesia workforce survey: twenty-year update. Anesthesiology..

[14] Carvalho B, Wang P, Cohen SE (2006). A survey of labor patient-controlled epidural anesthesia practice in California hospitals. Int J Obstet Anesth.

[15] Ninidze N, Bodin S, Ivester T, Councilman L, Clyne B, Owen M (2013). Advancing obstetric anesthesia practices in Georgia through clinical education and quality improvement methodologies. Int J Gynaecol Obstet.

[16] Kopic D, Sedensky M, Owen M (2009). The impact of a teaching program on obstetric anesthesia practices in Croatia. Int J Obstet Anesth.

[17] Sheiner E, Shoham-Vardi I, Ohana E, Segal D, Mazor M, Katz M. Characteristics of Parturients Who Choose to Deliver Without Analgesia. J Psychosom Obstet Gynaecol. 1999;20(3):165–9. 10.3109/01674829909075591 10497760

