# Correction to: Obstetric anesthesia services in Israel snapshot (OASIS) study: a 72 hour cross-sectional observational study of workforce supply and demand

**DOI:** 10.1186/s13584-021-00463-z

**Published:** 2021-04-06

**Authors:** Gal Schtrechman-Levi, Alexander Ioscovich, Jacob Hart, Jacob Bar, Ronit Calderon-Margalit, Eshel A. Nir, Yehuda Ginosar

**Affiliations:** 1 Department of Epidemiology and Preventive Medicine, School of Public Health, Sackler School of Medicine, Tel Aviv University, Tel Aviv-Yafo, Israel; 2 Department of General and Oncological Surgery – Surgery C, The Haim Sheba Medical Center, Ramat Gan, Israel; 3 Department of Anesthesiology, Perioperative Medicine, and Pain Treatment, Shaare Zedek Medical Center, and Faculty of Medicine, Hebrew University of Jerusalem, Jerusalem, Israel; 4 Health Services Management School, Netanya Academic College, Netanya, Israel; 5 Department of Obstetrics and Gynecology, Wolfson Medical Center, Holon, Israel; 6 Hadassah-Hebrew University Braun School of Public Health, Jerusalem, Israel; 7 Department of Anesthesia and Operating Rooms, Kaplan Medical Center, and Faculty of Medicine, Hebrew University of Jerusalem, Jerusalem, Israel; 8 Department of Anesthesiology and Critical Care Medicine, and Wohl Institute of Translational Medicine, Hadassah-Hebrew University Medical Center, and Faculty of Medicine, Hebrew University of Jerusalem, Jerusalem, Israel; 9 Department of Anesthesiology, Washington University School of Medicine, St Louis, MO USA

**Correction to: Isr J Health Policy Res 10, 24 (2021)**

**https://doi.org/10.1186/s13584-021-00460-2**

In the original publication of the article [1] there was a misunderstanding in the publication process which led to the missing hospital names in the **results and study limitations** section. The incorrect and correct information is shown here, the updated information is shown in **bold**. The original article has been updated.

**Incorrect**

- Twelve Israeli hospitals (7 governmental and 5 private) participated in the study: Shamir-Asaf HaRofe (Rishon Lezion), Poria (Tiberias), Mayanei Hayeshua (Bnei Brak), Ichilov (Tel Aviv), Wolfson (Holon), Barzilai (Ashkelon), Bnei Zion (Haifa), Hadassah Ein Karem (Jerusalem), Bikur Cholim (Jerusalem) and Shaarei Zedek (Jerusalem). Excluded hospitals included the seven hospitals of Clalit HMO: Rabin-Beilinson (Petach Tikva), Yoseftal (Eilat), Soroka (Beersheva), HaEmek (Afula), Meir (Kfar Saba), Kaplan (Rehovot), Carmel (Haifa). Also excluded were hospitals that did not obtain IRB approval: the Nazareth hospitals (English, Italian and French), Sheba (Tel Hashomer), Laniado (Netanya), Hillel Yaffe (Hadera), Rambam (Haifa).

**Correct**

- Twelve Israeli hospitals (7 governmental and 5 private) participated in the study: Shamir-Asaf HaRofe (Rishon Lezion), **Ziv (Tzfat)**, Poria (Tiberias), Mayanei Hayeshua (Bnei Brak), Ichilov (Tel Aviv), Wolfson (Holon), Barzilai (Ashkelon), Bnei Zion (Haifa), Hadassah Ein Karem (Jerusalem), **Hadassah Mt Scopus (Jerusalem)**, Bikur Cholim (Jerusalem) and Shaarei Zedek (Jerusalem). Excluded hospitals included the seven hospitals of Clalit HMO: Rabin-Beilinson (Petach Tikva), Yoseftal (Eilat), Soroka (Beersheva), HaEmek (Afula), Meir (Kfar Saba), Kaplan (Rehovot), Carmel (Haifa). Also excluded were hospitals that did not obtain IRB approval: the Nazareth hospitals (English, Italian and French), Sheba (Tel Hashomer), Laniado (Netanya), Hillel Yaffe (Hadera), Rambam (Haifa).

**Incorrect**

- There was some degree of geographical bias. The central region is represented reasonably appropriately; Shamir-Asaf HaRofe (Rishon Lezion), Mayanei Hayeshua (Bnei Brak), Ichilov (Tel Aviv), and Wolfson (Holon) are included, while Rabin-Beilinson (Petach Tikva), Meir (Kfar Saba), Sheba (Tel Hashomer), Laniado (Netanya) are not. However outside of the central region, there is geographical bias, with over-representation of Jerusalem and under-representation of the north and south. Clalit have no hospitals in the Jerusalem area, the highest birthrate area in the country, and so all the Jerusalem hospitals (Bikur Cholim, Hadassah Ein Karem and Hadassah Mt Scopus) were included. By comparison, in the north only Poria (Tiberias), Ziv (Tzfat), and Bnei Zion (Haifa) were included, while HaEmek (Afula), Carmel (Haifa), the Nazareth hospitals, Galilee Medical Center (Nahariya), Hillel Yaffe (Hadera), and Rambam (Haifa) were not. In the south only Barzilai (Ashkelon) was included, while Yoseftal (Eilat), Soroka (Beersheva), and Kaplan (Rehovot) were not.

**Correct**

- There was some degree of geographical bias. The central region is represented reasonably appropriately; Shamir-Asaf HaRofe (Rishon Lezion), Mayanei Hayeshua (Bnei Brak), Ichilov (Tel Aviv), and Wolfson (Holon) are included, while Rabin-Beilinson (Petach Tikva), Meir (Kfar Saba), Sheba (Tel Hashomer), Laniado (Netanya) are not. However outside of the central region, there is geographical bias, with over-representation of Jerusalem and under-representation of the north and south. Clalit have no hospitals in the Jerusalem area, the highest birthrate area in the country, and so all the Jerusalem hospitals (**Shaarei Zedek**, Bikur Cholim, Hadassah Ein Karem and Hadassah Mt Scopus) were included. By comparison, in the north only Poria (Tiberias), Ziv (Tzfat), and Bnei Zion (Haifa) were included, while HaEmek (Afula), Carmel (Haifa), the Nazareth hospitals, Galilee Medical Center (Nahariya), Hillel Yaffe (Hadera), and Rambam (Haifa) were not. In the south only Barzilai (Ashkelon) was included, while Yoseftal (Eilat), Soroka (Beersheva), and Kaplan (Rehovot) were not.
