# Supplementary material for: Obstetric anesthesia services in Israel snapshot (OASIS) study: a 72 hour cross-sectional observational study of workforce supply and demand
Source: Isr J Health Policy Res. 2021 Mar 15;10:24. doi: 10.1186/s13584-021-00460-2 (PMC7958695; doi:10.1186/s13584-021-00460-2)
Supplement: Supplementary file 1 — Additional file 1: Supplementary Table 1. Characteristics of hospitals that participated and that did not participate in the OASIS study. Data from Weiniger [2] and Shatalin [5] for years 2007 and 2018 respectively. [file 13584_2021_460_MOESM1_ESM.docx]

Supplementary Table 1. Characteristics of hospitals that participated and that did not participate in the OASIS study. Data from Weiniger [2] and Shatalin [5] for years 2007 and 2018 respectively.

|  | **Number deliveries  (total / cohort / year)** | | | **Number deliveries  (total / hospital / year)** | | | **Epidural rate (%)** | | | **Epidurals / year** | | |
| --- | --- | --- | --- | --- | --- | --- | --- | --- | --- | --- | --- | --- |
|  | **2018** | **2007** | **2007-2018 average** | **2018** | **2007** | **2007-2018 average** | **2018** | **2007** | **2007-2018 average** | **2018** | **2007** | **2007-2018 average** |
| Hospitals included (n=12) | 73,203 | 70,507 | 71,855 | 8,134 ± 4,603 | 6,013 ± 3,076 | 7,073 | 61  ± 16 | 53  ± 21 | 56 | 5,503 ± 3,034 | 3,383  ± 2,544 | 4,443 |
| Hospitals excluded  (n=15) | 79,307 | 75,720 | 77,513 | 6,609 ± 3,941 | 5,087 ± 3,180 | 5,848 | 56 ± 19 | 43  ± 24 | 48 | 3,610 ± 2,076 | 2,391  ± 2,239 | 3,001 |

|  | **Cesarean rate (%)** | | | **Cesareans / year** | | | **Obstetric anesthesia activity index (OAAI)** | | | **GA (%) for all cesareans** | **GA (%) for urgent cesareans** |
| --- | --- | --- | --- | --- | --- | --- | --- | --- | --- | --- | --- |
|  | **2018** | **2007** | **2007-2018 average** | **2018** | **2007** | **2007-2018 average** | **2018** | **2007** | **2007-2018 average** | **2018** | **2018** |
| Hospitals included (n=12) | 16  ± 5 | 19  ± 8 | 17.5 | 1,249  ± 713 | 1,049  ± 701 | 1,149 | 15.5  ± 9.0 | 10.0 ± 6.4 | 12.8 | 13  ± 8 | 21  ± 18 |
| Hospitals excluded  (n=15) | 21  ± 3 | 21  ± 4 | 21 | 1,336  ± 687 | 1,082 ± 724 | 1,209 | 12.9  ± 6.7 | 8.3 ± 6.2 | 10.6 | 21  ± 22 | 30  ± 31 |
